# Anti-Modified Peptide Antibodies (AMPAs) in Rheumatoid Arthritis: Study of the Diagnostic Value of Citrullinated, Homocitrullinated, and Acetylated Fibrin/Filaggrin Chimeric Peptides

**DOI:** 10.3390/diagnostics14222485

**Published:** 2024-11-07

**Authors:** Isabel Haro, Raul Castellanos-Moreira, Raimon Sanmartí, María José Gómara

**Affiliations:** 1 Unit of Synthesis and Biomedical Applications of Peptides, IQAC-CSIC, Jordi Girona 18–26, 08034 Barcelona, Spain; 2 Department of Rheumatology, Hospital Clínic of Barcelona, 08036 Barcelona, Spain; castellanos@clinic.cat (R.C.-M.); sanmarti@clinic.cat (R.S.)

**Keywords:** rheumatoid arthritis, anti-modified peptide antibodies, citrullination, homocitrullination, acetylation, synthetic peptides, fibrin, filaggrin, chimeric peptides

## Abstract

**Background/Objectives.** The presence of anti-citrullinated peptide/protein antibodies (ACPAs), anti-carbamylated peptide/protein antibodies (anti-CarPs), and anti-acetylated peptide/protein antibodies (AAPAs), collectively termed as anti-modified peptide/protein antibodies (AMPAs), is a hallmark of rheumatoid arthritis. These autoantibodies play a crucial role in the complex autoimmune responses observed in patients. Understanding the interplay between them is essential for early diagnosis and effective management of the disease. **Methods.** In this work, we investigate IgG, IgM, and IgA levels of ACPAs, anti-CarPs, and AAPAs in two cohorts: patients with established RA disease and healthy blood donors, using a unique peptide antigenic backbone. **Results.** Our results showed that antibody levels of anti-citrullinated peptide (CFFCP) and anti-homocitrullinated peptide (CFFHP) were significantly higher in RA patients compared to healthy blood donors in the three isotypes analyzed, IgG, IgA, and IgM. Fine specificities were more frequent when using the CFFCP antigen. Regarding the reactivity to the acetyl-lysine modified peptide (CFFAP), the correlation between IgA and IgG/IgM was very weak. CCFAP was highly specific for isotypes IgG and IgA, but its sensitivity was low for both isotypes. Anti-CarP and AAPA are significant in the context of RA, particularly concerning their IgA isotypes. **Conclusions.** Their inclusion in diagnostics assessments for RA, especially for anti-citrulline negative cases, presents a potential advance in the field; however, they do not replace yet traditional markers like rheumatoid factor (RF) and ACPAs.

## 1. Introduction

Rheumatoid arthritis (RA) is a very complex and heterogeneous chronic inflammatory disease affecting nearly 1% of general population [1,2]. Timely diagnosis of RA is crucial to optimizing long-term patient outcomes by preventing joint damage and disability. Therefore, identifying early disease is essential to initiate the treatment within a therapeutic window of opportunity, which may lead to better disease control and a reduction in work-related disability [3]. Although antibodies against citrullinated peptides/proteins (ACPAs), in which the post-translational modification (PTM) involved arginine residues in some proteins, are the autoantibodies currently employed in routine RA serodiagnostics [4,5,6,7], the presence of other autoantibodies in RA patients becomes of paramount importance. Anti-carbamylated protein antibodies (anti-CarPs) and anti-acetylated peptide antibodies (AAPAs) are directed against epitopes containing PTMs other than citrullination; anti-CarP antibodies being directed at homocitrullinated lysine residues, while AAPAs recognize epitopes in which lysine residues have been enzymatically modified to acetyl-lysine [8]. In the context of RA, the relationship between ACPA, anti-CarP, and AAPA antibodies is significant and each of these autoantibodies plays a crucial role in the complex autoimmune responses observed in RA [9]. Understanding the interplay between these autoantibodies, collectively termed as anti-modified protein/peptide antibodies (AMPAs), is essential for early diagnosis and effective management of rheumatoid arthritis [10].

The anti-Carp response [11,12,13] has been shown to be an independent risk factor for a severe extra-articular manifestation of RA that is associated with a significant increase in morbidity and mortality (interstitial lung disease, ILD) [14]. Therefore, early detection of anti-Carp response is crucial in establishing an individualized treatment strategy for these patients. The anti-CarP response initially occurs in the lungs of patients in an antigen-mediated manner, specifically involving the IgA isotype [15]. Moreover, the presence of AAPAs in RA has emerged as a significant area of research, shedding light on potential diagnostic advancements in the field, with recent articles indicating their potential value in early diagnosis and disease differentiation [16,17]. In this line of research, Studenic et al. [18] have shown that AAPAs are highly prevalent in early RA patients, even occurring independently of traditional markers like rheumatoid factor (RF) and ACPAs, thereby bridging a serological gap in diagnosis. The reactivity of ACPA and anti-CarP usually coincides. However, AAPA reactivity can be absent in ACPA-positive subjects, which seems to indicate that AAPAs belong to a different category of AMPAs.

Although most studies on AMPAs’ reactivity are centered on the presence of IgG autoantibodies, the relevance of the determination of fine specificity has been increasingly reported since it may indeed provide valuable additional clinical information of RA patients [7]. In this sense, the IgM isotype which is the first antibody generated in the autoimmune response could be the starting point of the AMPA responses associated with RA, after which isotype and epitope spreading would occur giving rise to the typical AMPA response associated to the RA [19]. On the other hand, IgA has been related to the pathophysiology of RA, inducing the formation of neutrophil extracellular traps (NETs) more potently than IgG complexes [20] which might contribute to the onset and perpetuation of the disease through exposure to autoantigens [21].

In this work, we investigate IgG, IgM, and IgA levels of ACPA, anti-CarP, and AAPA in two cohorts: patients with established RA disease and healthy blood donors, using the same peptide antigenic backbone for the three PTMs: a cyclic chimeric fibrin/filaggrin peptide previously designed and studied in our research group in citrullinated and homocitrullinated versions [14,22,23], which showed high sensitivity and specificity for RA in comparison with healthy controls and those with other chronic diseases such as patients with systemic lupus erythematosus (SLE) according to ACR criteria, patients with psoriatic arthritis (PsA) fulfilling the Wright and Moll criteria, and patients with chronic hepatitis C virus (HCV) infection (confirmed with nucleic acid testing and antibody test results). Our aim is to provide some insights to contribute to the understanding of the development of autoantibodies, the expansion of anti-modified peptide antibodies, and the isotype spectrum in RA patients which is essential for unraveling the complex mechanisms underlying the autoimmune response in this devastating disease.

## 2. Materials and Methods

### 2.1. Synthesis of Fibrin/Filaggrin Chimeric Peptides: CFFCP, CFFHP, and CFFAP

**Figure 1:**
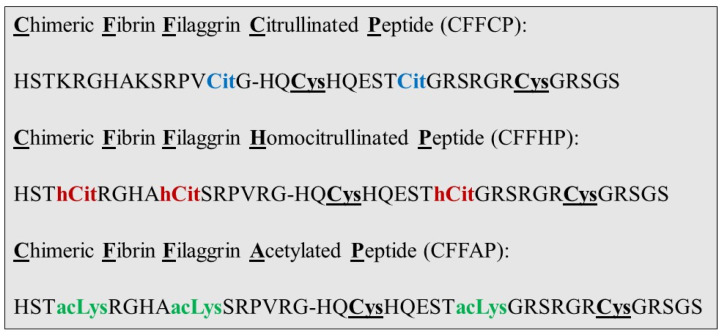
Primary structures of chimeric fibrin/filaggrin peptides. An intramolecular cyclisation was established between the Cys residues (highlighted in bold and underlined).

CFFCP: [Cit^630^] α-fibrin (617–631)-*S*^306^, *S*^319^ *cyclo* [Cys^306,319^, Cit^312^] filaggrin (304–324); CFFHP: [hCit^620,625^] α-fibrin (617–631)-*S*^306^, *S*^319^ *cyclo* [Cys^306,319^, hCit^312^] filaggrin (304–324); and CFFAP: [acLys^620,625^] α-fibrin (617–631)-*S*^306,S319^ *cyclo* [Cys^306,319^, acLys^312^] filaggrin (304–324) were synthesized manually in solid phase, following procedures previously described [3,10]. Final products were purified via semi-preparative HPLC (Agilent Technologies 1260 Infinity chromatograph, Santa Clara, CA, USA) on a Kromasil C-18 column 5 μm, 25 × 1 cm (Tecknokroma, Barcelona, Spain) with a linear gradient of 100–75% A in B over 30 min at a flow rate of 4 mL/min using 0.05% TFA in water (A) and 0.05% TFA in acetonitrile (B) as an eluting system. The purified peptides (purity higher than 95%) were characterized using analytical UPLC (Waters Acquity, Waters corporation, Mildford, MA, USA) and ES-MS (Waters LCT Premier XE, Micromass Waters, Mildford, MA, USA) (Figures S1–S3). The primary structure of CFFCP, CFFHP, and CFFAP is indicated in Figure 1.

### 2.2. Serum Samples

A cross-sectional study including 178 patients with RA according to the 2010 ACR/EULAR criteria and 120 sera from healthy blood donors (BD) was carried out. All consecutive patients with established RA and assessed between July 2017 and July 2018 in the outpatient clinic in a Rheumatology department of a tertiary university hospital were included. Patients fulfilling other inflammatory rheumatic diseases diagnostic criteria were excluded. Demographic, clinical, and laboratory characteristics of patients at study entry are shown in Table 1.

### 2.3. ELISA Assays

**Figure 2:**
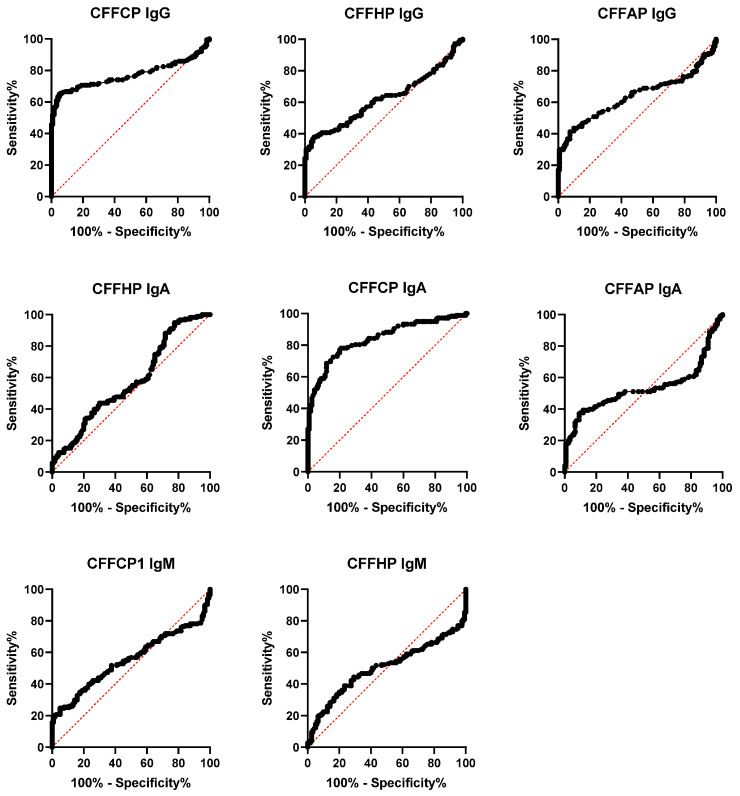
Receiver operating curves (ROCs) of IgG, IgA, and IgM antibodies against CFFCP, CFFHP, and CFFAP in RA patients versus healthy blood donors.

The ELISA assays were carried out following procedures previously described [14]. In brief, MaxiSorp microtiter plates were incubated with Neutravidin protein diluted in PBS overnight at 4 °C and, thereafter, 1 h at 37 °C. The biotinyl-peptides were diluted at 1 μg/mL in PBS, and 100 μL of the peptide solution was added to each well. Sera were diluted 250-fold in RIA buffer supplemented with 10% fetal bovine serum, and 100 μL of the dilution was added to each well. The plates were incubated for 1 h at 37 °C and then overnight at 4 °C. After washing the plates, 100 μL of anti-human IgG or anti-human IgA or anti-human IgM secondary antibodies conjugated to peroxidase were diluted in RIA buffer at 1:4000 for IgG, 1:2000 for IgA, or 1:10,000 for IgM and were added to each well and incubated for 1 h at 37 °C. The detection of bound antibodies was carried out using SigmaFast (Sigma-Aldrich, St. Louis, MO, USA), with *o*-phenylenediamine dihydrochloride (OPD) as a substrate. All sera were tested in duplicate. Reactivity to the unmodified basal peptide structures (CFFP; all arginine and lysine) was subtracted from the reactivity to CFFCP, CFFHP, and CFFAP, respectively, to ensure that the measured reactivity shown was specific to the post-translational modification under study. Results were obtained in values of optical density (OD) and ranged from 0 to 4, where 0 implies no reactivity and 4 is the highest value of intensity of reactivity. ROC curves were obtained and shown in Figure 2.

### 2.4. Statistical Analysis

Differences in autoantibody levels between groups were calculated with Mann–Whitney tests and correlation coefficients with Spearman r. Analyses were performed in Graph Pad Prim 8.0.2.

## 3. Results

### 3.1. Synthesis of the Peptide Antigens

The three chimeric peptides, whose primary structures are shown in Figure 1, were successfully synthesized and conveniently labeled at the N-terminus with Biotinyl-PEG_2_ following Solid Phase Peptide Synthesis (SPPS) protocols as previously described [15]. The introduction of this spacer ensures a good exposure of the peptide antigens when they are bounded to the MaxiSorp Neutravidin microtiter plates. Moreover, a control peptide (CFFP: HSTKRGHAKSRPVRG-HQCysHQESTRGRSRGRCysGRSGS) was also synthesized and biotinylated at the N-terminus via SPPS to subtract its reactivity against the sera from the reactivity obtained with CFFCP, CFFHP, and CFFAP and thus to ensure that the measured reactivity was specific to the PTM under study.

### 3.2. Autoantibodies Status in RA Patients

**Figure 3:**
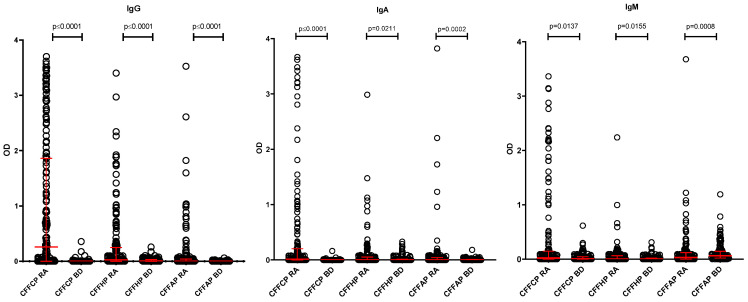
IgG, IgA, and IgM antibodies’ reactivity to CFFCP, CFFHP, and CFFAP peptides in RA patients and healthy blood donors. Differences in antibody levels between groups were calculated with Mann–Whitney test. Medians with IQR are indicated in red.

A comparative ELISA assay was performed in cohorts of RA and BD sera using CFFCP, CFFHP, and CFFAP as coating antigens; as it has been described in 2.3. The experimental ROC curves (RA vs. BD) are illustrated in Figure 2, and the values of the area under (AUC) obtained are indicated in Table 2, where the sensitivity and specificity values obtained at optical density ≥0.1 are also shown. Antibody levels for anti-CFFCP and anti-CFFHP were significantly higher in RA patients compared to the healthy blood donors in the three isotypes analyzed (Figure 3). Of note, IgG, IgA, and IgM fine specificities were more frequent when using the citrullinated antigen (CFFCP), and the IgM anti-CFFAP could not be determined due to the high levels of this isotype in the HD cohort.

We compared the sensitivity and specificity of the different PTMs using the same peptide structure with a cut-off ≥ 0.1 in optical density (Table 2). Thus, to consider serum positivity, the difference between the reactivity of sera to peptides with PTMs (CFFCP, CFFHP, and CFFAP) and the unmodified basal peptide structure (with arginine and lysine) must always be equal to or greater than 0.1 in optical density. At this cut-off, the three modified peptides showed high specificity (>95% for CFFHP IgG; >88% for CFFHP IgA; and >86% for CFFCP IgM) but with different sensitivities being the highest for the citrullinated peptide. The acetylated peptide was highly specific for isotypes IgG and IgA (>100% and >99.17%, respectively) but its sensitivity was low for both isotypes (14.61% and 11.24%, respectively). A cut-off for establishing anti-CFFAP IgM positivity could not be calculated due to the high levels of anti-CFFAP IgM in healthy blood donors.

### 3.3. Correlation of Antibodies Anti-CFFCP, Anti-CFFHP, and Anti-CFFAP with Rheumatoid Factor (RF)

The correlation of the different studied AMPAs and isotypes with other traditional markers like RF has also been studied. We measured the serum RF via nephelometry, as in our clinical practice, which only measures the IgM isotype. As expected and as it is shown in Table 3, an association between RF and the different AMPAs was found. The only exception was the IgM isotype for which the results obtained were non-significant for the two antigens (CFFCP and CFFHP) under study. As indicated previously, the IgM anti-CFFAP could not be analyzed as a consequence of the high level of these antibodies that were found in the BD panel of sera.

### 3.4. Correlation Between Antibodies Anti-CFFCP, Anti-CFFHP, and Anti-CFFAP

**Figure 4:**
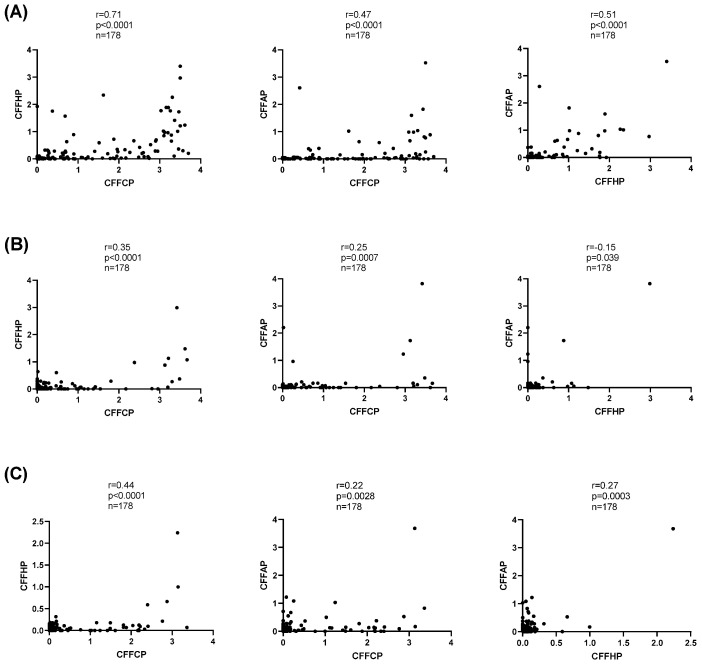
Spearman correlation between citrullinated, homocitrullinated, and acetylated peptide-based immunoassays (**A**): IgG, (**B**): IgA, and (**C**): IgM.

**Figure 5:**
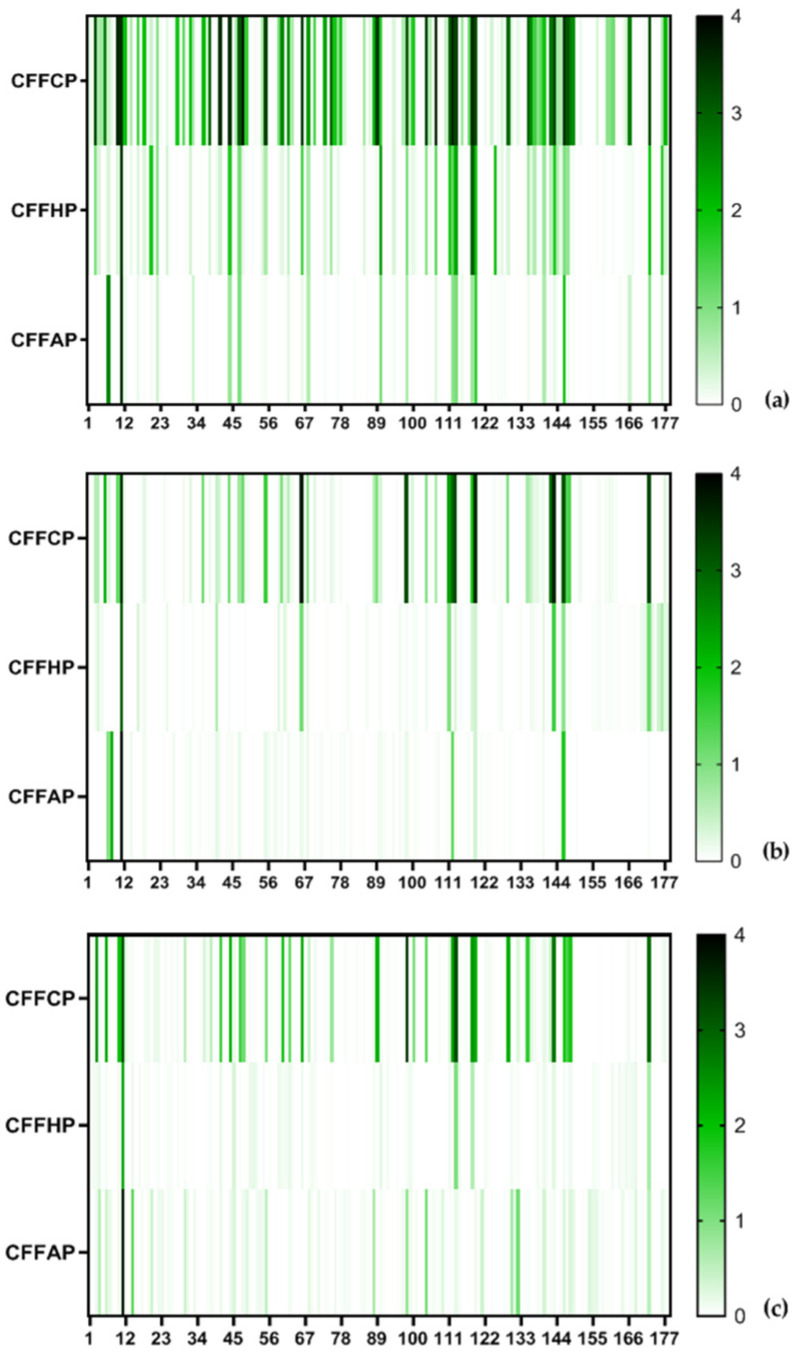
Heat map of the IgG (**a**), IgA (**b**), and IgM (**c**) reactivity in optical density (OD) of RA patient’s sera to peptides containing citrulline or homocitrulline or acetyl-lysine.

When analyzing the correlation between the levels of antibodies’ anti-modified peptides, we observed that in general for the three isotypes, the correlation between anti-citrulline and anti-homocitrulline reactivity was higher than the correlation between the reactivity to these PTMs and anti-acetyl-lysine (Figure 4). IgG anti-homocitrulline (CFFHP) and anti-acetyl-lysine (CFFAP) reactivity was mainly observed in RA sera with high anti-citrulline (CFFCP) reactivity (Figure 4A). However, not all patients with high anti-CFFCP levels also have anti-CFFHP and anti-CFFAP reactivity as can be observed in the heat map of IgG antibody reactivities of RA patients’ sera to PTM modified peptides (Figure 5). Particularly, IgG anti-CFFAP was present only in a smaller subset of anti-CFFCP reactive patients.

Regarding the IgA isotype, correlation between modified peptides were weak, and, specifically, the IgA correlation between reactivity to the CFFAP and CFFHP showed a low negative Spearman r (Figure 4B).

**Figure 6:**
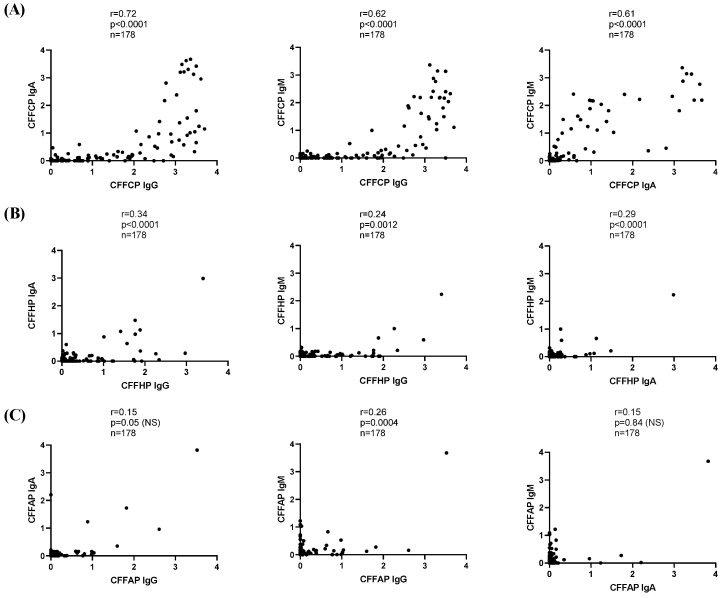
Spearman correlation between IgG, IgA, and IgM isotypes of (**A**): CFFCP, (**B**): CFFHP, and (**C**): CFFAP.

The assays performed with the different PTMs presented at the same peptide backbone enabled a direct comparison between IgG, IgA, and IgM AMPA reactivities. We also observed a stronger correlation between IgG and IgA/IgM reactivity to the citrulline modified peptide (CFFCP) than to the homocitrulline peptide (CFFHP) (Figure 6A,B). Thus, IgA and IgM reactivity to CFFCP was primarily detected in individuals with high levels of IgG reactivity to CFFCP (Figure 6A). On the contrary, the correlation between IgA and IgG/IgM reactivity to the acetyl-lysine modified peptide (CFFAP) was very weak suggesting a distinct pattern found in IgA anti CFFAP (Figure 6C).

### 3.5. Serological Classification Based on Antibody Concordance

**Figure 7:**
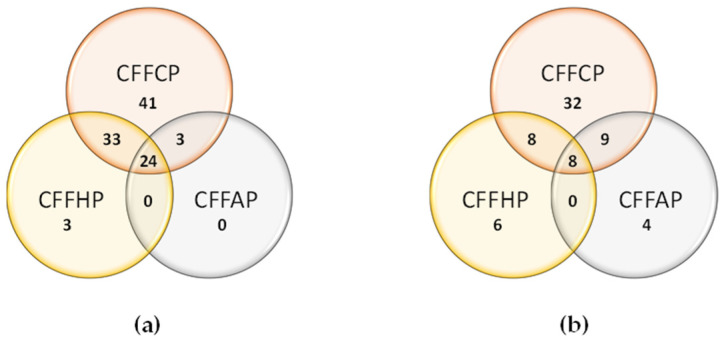
Venn diagram for positivity among (**a**): IgG and (**b**): IgA anti-citrulline, anti-homo-citrulline, and anti-acetyl-lysine reactivity in 178 RA patient’s sera.

As shown in Figure 7, there was a considerable overlap between the IgG reactivity to the different PTMs presented by the peptides. Almost all IgG anti-homocitrulline-positive patients were IgG anti-citrulline-positive. Concerning IgG anti-acetyl-lysine-positive patients, all sera were also positive for citrulline. Regarding IgA reactivity, less overlapping was observed between reactivity to the different peptides (Figure 7b). There was 9% (6/67) and 6% (4/67) of IgA positive RA sera that only reacted to CFFHP and CFFAP, respectively.

## 4. Discussion

The relationship between ACPA, anti-CarP, and AAPA antibodies in RA is significant, and these autoantibodies appear to play a crucial role in the complex autoimmune responses observed in this autoimmune disease. It has been previously described that immunization of mice with a specific post-translationally modified protein provides an anti-modified protein antibody response that recognizes different post-translationally modified antigens [24]. The evolution of AMPA responses can be driven by consecutive exposure to different PTMs. However, it is unclear whether the AMPA reactivity profile is “fixed” in time or whether consecutive exposure to different post-translationally modified proteins can shape the evolving AMPA response toward a specific post-translationally modified protein. Serum samples collected longitudinally from individuals at risk for RA and those with early RA have shown dynamic changes in AMPA reactivity profiles [25].

To measure AMPA reactivity in RA patients with a well stablished disease, our approach involves peptide domains selected from different proteins that allow the presentation of diverse epitopes to the immune system. The chimeric peptide antigenic backbones studied in the present work have been previously engineered [15,22] to offer a versatile platform for creating tailored antigens with properties for immunological applications and specifically to detect autoantibodies in RA patients. We compared the balance of sensitivity/specificity as well as the correlation between the levels of antibodies’ anti-modified peptides for the three isotypes, using the same peptide chemically well-defined antigen to directly perform the comparison by using the appropriate native peptide control. This approach ensures consistent and reproducible testing of autoantibody specificities and can comprehensively capture the diverse autoantibody repertoire in RA patients.

We could observe that anti-homocitrulline (anti-CFFHP) and anti-acetyl-lysine (anti-CFFAP) reactivity primarily coexisted with citrulline reactivity (anti-CFFCP), and in spite of working with different peptide antigens, our results were in agreement with the data published by Gronwall et al. [26]. On the other hand, high levels of anti-CFFAP IgM were detected in healthy blood donors. This result is in accordance with the results recently published by van Wesemael et al. [19] suggesting that IgM antibodies against acetylated proteins is part of the “normal” immune repertoire and might possibly constitute a starting point for RA-associated AMPA responses.

Comparatively to the IgG isotype, there was less concordance in IgA reactivity between the three peptides and there was a 9% and a 6% rate of IgA-positive sera that only recognized the homocitrulline and acetyl-lysine modifications, respectively. The low concordance in IgA reactivity between peptides containing citrulline, homocitrulline, and acetyl-lysine modifications indicates that IgA antibodies in RA can have distinct specificity profiles and suggest that different post-translational modifications (PTMs) may trigger unique IgA responses. These results emphasize the significance of using chemically well-defined peptides that incorporate all three PTMs (citrulline, homocitrulline, and acetyl-lysine) on the same backbone. This approach might be crucial for comprehensively identifying IgA reactivity in RA patients providing insights into RA pathogenesis.

As previously reported, the classification of RA patients based on serological profiles, particularly focusing on antibody concordance among anti-citrulline, anti-homocitrulline, and anti-acetylated antigens, has shown promising advancements. Regueiro et al. emphasized the importance of the concordance of multiple autoantibodies, including RF, anti-citrullinated, and anti-CarP antibodies, in improving the specificity and accuracy of RA classification [27]. In our hands, antibody levels of anti-citrullinated peptide (CFFCP) and anti-homocitrullinated peptide (CFFHP) were significantly higher in RA patients compared to the healthy blood donors in the three isotypes analyzed, with IgG, IgA, and IgM fine specificities being more frequent when using the CFFCP antigen. However, the correlation between IgA and IgG/IgM reactivity to the acetyl-lysine-modified peptide (CFFAP) was very weak. CCFAP was highly specific for isotypes IgG and IgA, but its sensitivity was low for both isotypes.

Exploring the relationship between mucosal antibodies (e.g., salivary IgA) and circulating antibodies in the context of the mucosal origin hypothesis of RA [28] seem to be crucial for understanding how RA-related autoimmunity may originate at mucosal sites before becoming systemic. This knowledge could inform strategies for early detection and prevention of RA by targeting mucosal processes. In the last years, new evidence has been generated about the role of the mucosal involvement in the initiation and perpetuation of the rheumatoid process. It has been suggested that localized autoimmunity, mainly in the bronchial mucosa, may be crucial in the development of further systemic autoimmunity and the development of clinical RA [29]. Several studies confirm the presence of immune-cell activation in the mucosal bronchial tissue in patients with early RA even without lung disease. Furthermore, ACPA are found in the sputum and bronchi alveolar lavage in patients with RA but also in ACPA positive individuals at risk for RA progression, confirming the local secretion of these autoantibodies in the respiratory mucosa [30]. In fact, the presence of ACPA secreting plasma blasts has been confirmed in this population, suggesting that the selection of RA-specific autoantibody-positive B cells may occur in the lungs [31].

To address the prognostic value of different antibodies and isotypes, future studies should focus on comparing the prognostic value of different antibody isotypes and investigate the combined predictive power of multiple antibodies. In conclusion, serological classification based on antibody concordance represents a significant advancement in the diagnostic approach to rheumatoid arthritis, offering a more reliable and specific method for identifying affected individuals. This approach not only enhances the accuracy of RA classification but also holds potential for improving patient management and outcomes.

## Figures and Tables

**Table 1:** Demographic, clinical, and therapeutic characteristics of RA patients.

Female (%)	141 (79)
Age mean (±SD)	59.7 (13.0)
Mean disease duration (±SD)	6.6 (5.0)
Ever smokers (%)	83 (46)
Caucasian (%)	151 (84)
Rheumatoid factor positive	111 (62)
Extra-articular manifestations	
Sicca syndrome	33 (18)
Rheumatoid nodules	21 (12)
Serositis	3 (2)
Treatment	
Glucocorticoids (%)	108 (60)
csDMARDs (%)	155 (87)
MTX (%)	115 (64)
bDMARDs (%)	47 (26)
Mean DAS28 (±SD)	2.94 (1.18)
Radiographic erosive disease (%)	89 (50)

csDMARDS: conventional synthetic disease-modifying antirheumatic drugs, MTX: methotrexate, bDMARDS: biological DMARDS. DAS: disease activity score.

**Table 2:** Sensitivity and specificity at the cut-off at an optical density ≥0.1 and AUC (95% CI) of the ROC curves.

Peptides (IgG)	Sensitivity	95% CI	Specificity	95% CI	LR	AUC
CFFCP	56.74	49.40–63.80	98.33	94.13–99.70	34.0	0.771 (0.717–0.826)
CFFHP	32.02	25.61–39.19	95.83	90.62–98.21	7.68	0.622 (0.560–0.685)
CFFAP	14.61	10.17–20.54	100	96.90–100	-	0.637 (0.575–0.699)
**Peptides (IgA)**	**Sensitivity**	**95% CI**	**Specificity**	**95% CI**	**LR**	**AUC**
CFFCP	32.02	25.61–39.19	99.17	95.43–99.96	38.4	0.841 (0.797–0.885)
CFFHP	15.17	10.64–21.17	88.33	81.37–92.92	1.30	0.577 (0.510–0.643)
CFFAP	11.24	7.39–16.72	99.17	95.43–99.96	13.5	0.533 (0.467–0.599)
**Peptides (IgM)**	**Sensitivity**	**95% CI**	**Specificity**	**95% CI**	**LR**	**AUC**
CFFCP	26.40	20.48–33.33	86.67	79.44–91.62	1.98	0.553 (0.488–0.617)
CFFHP	16.29	11.59–22.42	94.17	88.45–97.15	2.79	0.501 (0.436–0.566)
CFFAP	ND	ND	ND	ND	ND	ND

AUC, area under the curve; CI, confidence interval; LR, likelihood ratio; ND, not determined.

**Table 3:** AMPAs status in RA patients (RF+ vs. RF−).

	RF+ (*n* = 110)	RF− (*n* = 68)	*p* Value
IgG			
anti-CFFCP+ (%)	75 (68.2)	26 (38.2)	*p* < 0.0001
median titer anti-CFFCP (IQR)	0.683 (2.613)	−0.007 (0.583)	*p* < 0.0001
anti-CFFHP+ (%)	49 (44.5)	11 (16.2)	0.0001
median titer anti-CFFHP (IQR)	0.069 (0.378)	0.004 (0.039)	*p* < 0.0001
anti-CFFAP+ (%)	24 (21.8)	3 (4.4)	0.0017
median titer anti-CFFAP (IQR)	0.005 (0.056)	−0.011 (0.013)	0.0043
IgA			
anti-CFFCP+ (%)	49 (44.5)	8 (11.8)	*p <* 0.0001
median titer anti-CFFCP (IQR)	0.038 (0.579)	−0.005 (0.035)	0.0005
anti-CFFHP+ (%)	18 (16.4)	4 (5.9)	0.039
median titer anti-CFFHP (IQR)	−0.024 (0.068)	−0.055 (0.028)	NS
anti-CFFAP+ (%)	18 (16.4)	3 (4.4)	0.016
median titer anti-CFFAP (IQR)	0.004 (0.052)	−0.016 (0.037)	NS
IgM			
anti-CFFCP+ (%)	31 (28.2)	17 (25)	NS
median titer anti-CFFCP (IQR)	0.032 (0.158)	0.010 (0.134)	NS
anti-CFFHP+ (%)	20 (18.2)	6 (8.8)	NS
median titer anti-CFFHP (IQR)	0.001 (0.082)	−0.042 (0.047)	NS

NS, Non-significant.

## Data Availability

Data are contained within the article or Supplementary Materials. The data presented in this study are available on request from the corresponding author.

## Supplementary Materials

The following supporting information can be downloaded at: https://www.mdpi.com/article/10.3390/diagnostics14222485/s1, Figure S1: Characterization of CFFCP via HPLC and ESI-MS; Figure S2: Characterization of CFFHP via HPLC and ESI-MS; Figure S3: Characterization of CFFAP via HPLC and ESI-MS.
