# Supplementary material for: Anti-Modified Peptide Antibodies (AMPAs) in Rheumatoid Arthritis: Study of the Diagnostic Value of Citrullinated, Homocitrullinated, and Acetylated Fibrin/Filaggrin Chimeric Peptides
Source: Diagnostics (Basel). 2024 Nov 7;14(22):2485. doi: 10.3390/diagnostics14222485 (PMC11593125; doi:10.3390/diagnostics14222485)
Supplement: Supplementary file 1 [file diagnostics-14-02485-s001.zip › diagnostics-3232916-supplementary.pdf]

Anti-Modified Peptide Antibodies (AMPAs) in Rheumatoid Arthritis: Study of the Diagnostic Value of Citrullinated, Homocitrullinated, and Acetylated Fibrin/Filaggrin Chimeric Peptides

Isabel Haro [1,*], Raul Castellanos-Moreira [2], Raimon Sanmartí [2] and María José Gómara [1,*]

[1] Unit of Synthesis and Biomedical Applications of Peptides, IQAC-CSIC, Jordi Girona 18–26, 08034 Barcelona, Spain
[2] Department of Rheumatology, Hospital Clínic of Barcelona, 08036 Barcelona, Spain; castellanos@clinic.cat (R.C.-M.); sanmarti@clinic.cat (R.S.)
* Correspondence: isabel.haro@iqac.csic.es (I.H.); mariajose.gomara@iqac.csic.es (M.J.G.)

Supplementary Material:

Figure S1: Characterization of CFFCP by HPLC and ESI-MS

Figure S2: Characterization of CFFHP by HPLC and ESI-MS

Figure S3: Characterization of CFFAP by HPLC and ESI-MS

A)

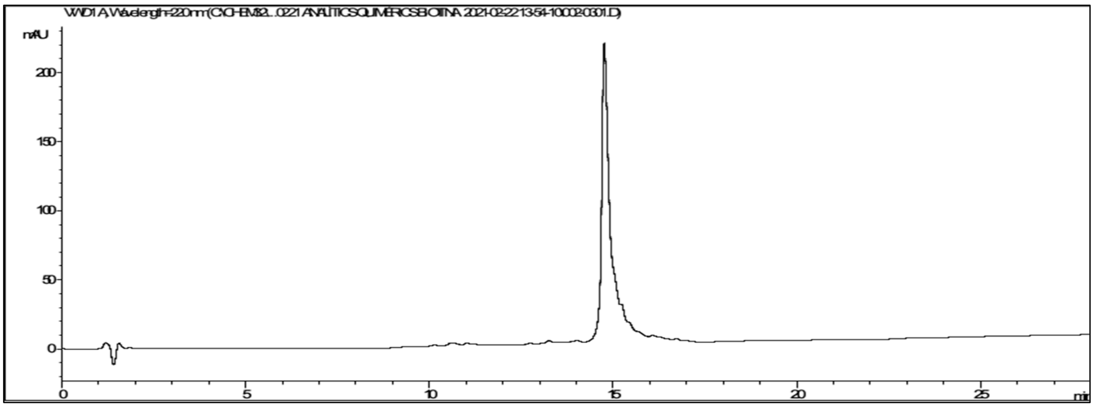

B)

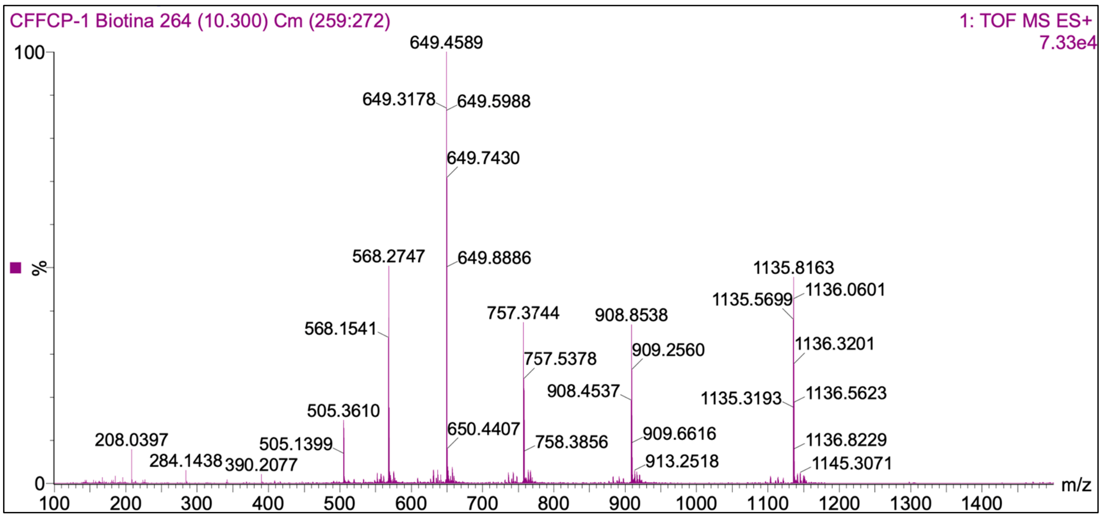

Figure S1. Characterization of CFFCP by HPLC and ESI-MS. (A) HPLC was performed on an Eclipse Plus C18 column (Agilent, 3.5 µm, 4.6 × 100 mm) with a linear gradient (5%-95%) of 0.05% TFA in ACN (solvent B) into 0.05% TFA in water (solvent A) over 30 min at 1 mL/min flow rate. (B) The mass spectrum was recorded in positive ion mode in the m/z 100-1500 range. LRMS: calculated m/z for $C_{181}H_{305}N_{75}O_{57}S_3$: 1136.01 $[M+4H]^{4+}$, 909,01 $[M+5H]^{5+}$, 757.68 $[M+6H]^{6+}$, 649,58 $[M+7H]^{7+}$, 568,51 $[M+8H]^{8+}$, 505.45 $[M+9H]^{9+}$; found by MS (ESI): 1135.82 $[M+4H]^{4+}$, 908,85 $[M+5H]^{5+}$, 757,37 $[M+6H]^{6+}$, 649,46 $[M+7H]^{7+}$, 568,27 $[M+8H]^{8+}$, 505.36 $[M+9H]^{9+}$.

A)

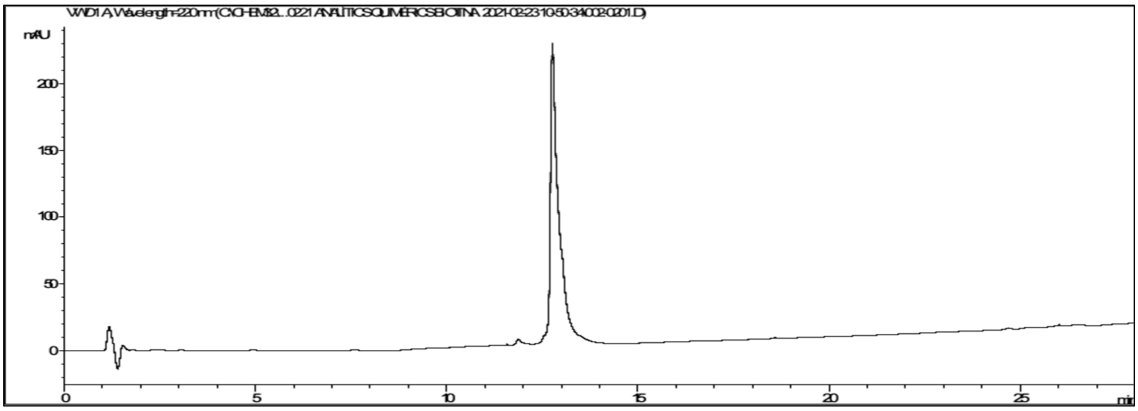

B)

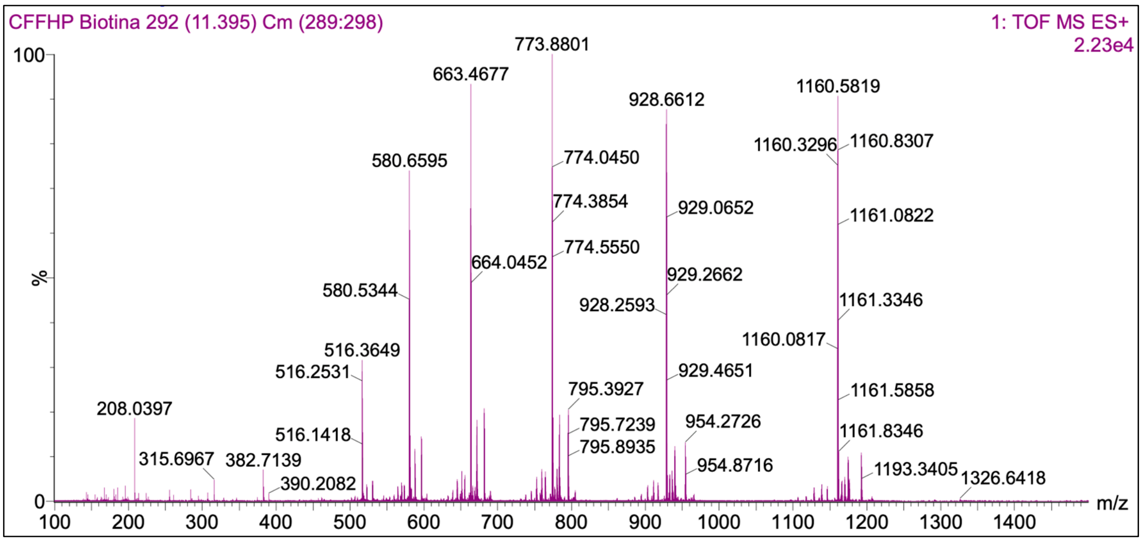

Figure S2. Characterization of CFFHP by HPLC and ESI-MS. (A) HPLC was performed on an Eclipse Plus C18 column (Agilent, 3.5 µm, 4.6 × 100 mm) with a linear gradient (5%-95%) of 0.05% TFA in ACN (solvent B) into 0.05% TFA in water (solvent A) over 30 min at 1 mL/min flow rate. (B) The mass spectrum was recorded in positive ion mode in the m/z 100-1500 range. LRMS: calculated m/z for $C_{184}H_{310}N_{78}O_{58}S_3$: 1160.79 $[M+4H]^{4+}$, 928.83 $[M+5H]^{5+}$, 774.19 $[M+6H]^{6+}$, 663.74 $[M+7H]^{7+}$, 580.90 $[M+8H]^{8+}$, 516.46 $[M+9H]^{9+}$; found by MS (ESI): 1160.58 $[M+4H]^{4+}$, 928.66 $[M+5H]^{5+}$, 773.88 $[M+6H]^{6+}$, 663.46 $[M+7H]^{7+}$, 580.66 $[M+8H]^{8+}$, 516.36 $[M+9H]^{9+}$.

A)

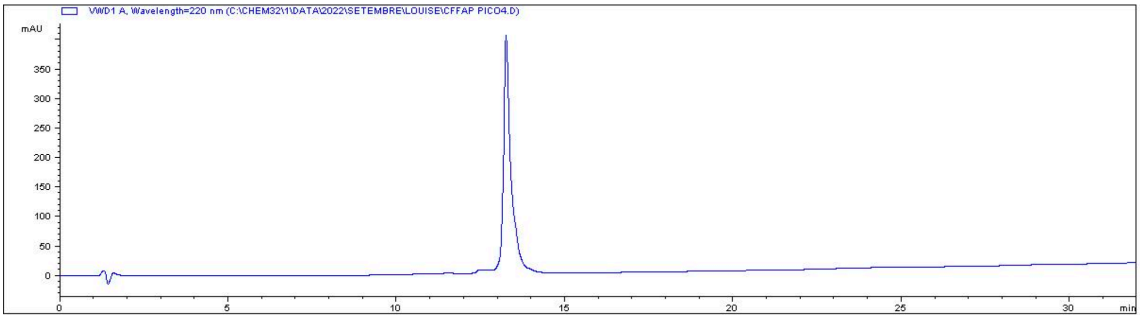

B)

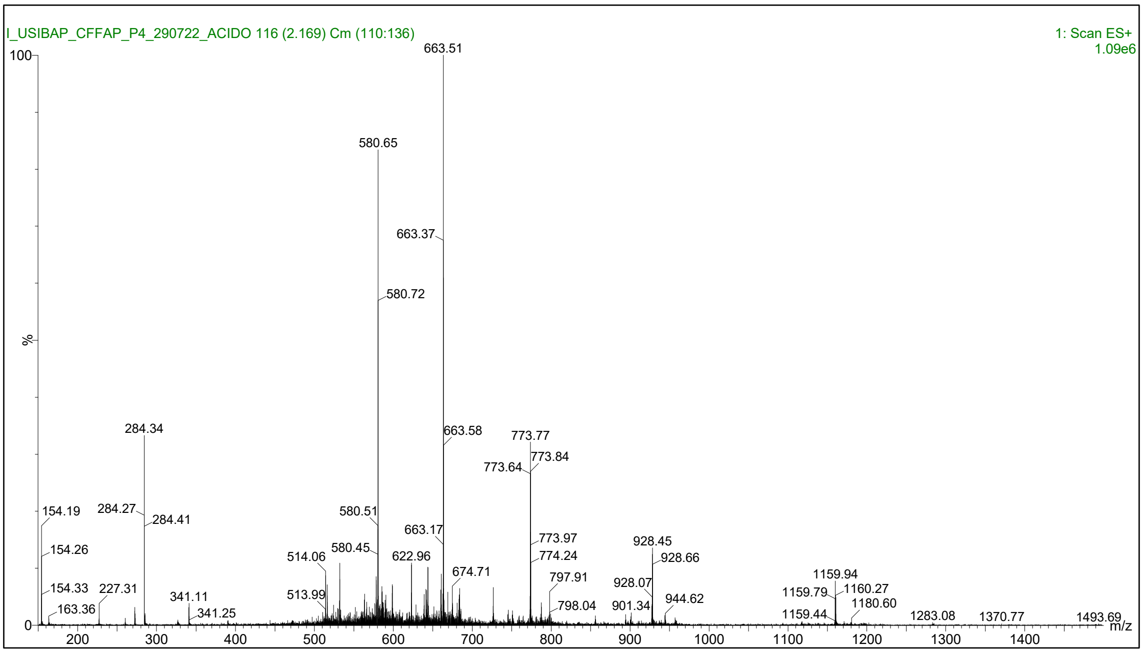

Figure S3. Characterization of CFFAP by HPLC and ESI-MS. (A) HPLC was performed on an Eclipse Plus C18 column (Agilent, 3.5 µm, 4.6 × 100 mm) with a linear gradient (5%-95%) of 0.05% TFA in ACN (solvent B) into 0.05% TFA in water (solvent A) over 30 min at 1 mL/min flow rate. (B) The mass spectrum was recorded in positive ion mode in the m/z 100-1500 range. LRMS: calculated m/z for $C_{187}H_{313}N_{75}O_{58}S_3$: 1160.04 $[M+4H]^{4+}$, 928.34 $[M+5H]^{5+}$, 773.70 $[M+6H]^{6+}$, 663.31 $[M+7H]^{7+}$, 580.53 $[M+8H]^{8+}$; found by MS (ESI): 1159.94 $[M+4H]^{4+}$, 928.45 $[M+5H]^{5+}$, 773.77 $[M+6H]^{6+}$, 663.51 $[M+7H]^{7+}$, 580.65 $[M+8H]^{8+}$.
